# Temperature-Dependent Carrier Transport in GaN Nanowire Wrap-Gate Transistor

**DOI:** 10.3390/nano13101629

**Published:** 2023-05-12

**Authors:** Siva Pratap Reddy Mallem, Peddathimula Puneetha, Yeojin Choi, Seung Mun Baek, Sung Jin An, Ki-Sik Im

**Affiliations:** 1 Advanced Material Research Center, Kumoh National Institute of Technology, Gumi 39177, Republic of Korea; drmspreddy@kumoh.ac.kr; 2 Department of Robotics and Intelligent Machine Engineering, College of Mechanical and IT Engineering, Yeungnam University, Gyeongsan 38541, Republic of Korea; puneethaphd@gmail.com; 3 Department of Materials Science and Engineering, Kumoh National Institute of Technology, Gumi 39177, Republic of Korea; dota23@kumoh.ac.kr (Y.C.); monndal980@kumoh.ac.kr (S.M.B.); 4 Department of Green Semiconductor System, Daegu Campus, Korea Polytechnics, Daegu 41765, Republic of Korea

**Keywords:** wrap-gate transistor, nanowire, GaN, surface/core, carrier scattering

## Abstract

For the creation of next-generation nanoscale devices, it is crucial to comprehend the carrier transport mechanisms in nanowires. Here, we examine how temperature affects the properties of GaN nanowire wrap-gate transistors (WGTs), which are made via a top-down technique. The predicted conductance in this transistor remains essentially unaltered up to a temperature of 240 K and then increases after that as the temperature rises. This is true for increasing temperature at gate voltages less than threshold voltage (*V*_gs_ < *V*_th_). Sharp fluctuations happen when the temperature rises with a gate voltage of *V*_th_ < *V*_gs_ < *V*_FB_. The conductance steadily decreases with increasing temperature after increasing the gate bias to *V*_gs_ > *V*_FB_. These phenomena are possibly attributed to phonon and impurity scattering processes occurring on the surface or core of GaN nanowires.

## 1. Introduction

Due to their potential usage in next-generation, high-performance electronic and optoelectronic devices, nanoscale circuit elements such as semiconductor-based nanowires have undergone significant development [1,2,3,4,5,6,7,8,9,10,11,12]. For example, nanowires can significantly shrink field-effect transistor (FET) geometries. As with FET, Fin-FET, tri-gate, omega gate, and gate-all-around (GAA) or wrap-gate (WG) devices, the scaling of various transistor types depends on geometries [13,14,15,16,17,18,19,20,21]. Due to their superior electrostatic controls, WG-based devices produce exceptionally impressive results when compared to alternative architectures. In comparison to the conventional technique (i.e., bottom-up), top-down device production of nanowire-based WG transistors (WGTs) has many advantages, including reduced device size, an orderly alignment of nanowires in a parallel pattern, and a high yield is attainable at a large scale. Device performance in high-speed, high-power, high-frequency, and high-temperature applications is superior for GaN-based nanowire devices. With a high *I*_on_/*I*_off_ ratio, a tiny gate leakage, and a high conductance, a GaN nanowire-based device can regulate its normally-off state. This work involved the top-down fabrication of a device and an investigation of the carrier transport mechanism in a GaN nanowire WGT on a GaN-on-insulator (GaNOI) substrate.

The operation of nanowire devices at cryogenic temperatures is particularly interesting since, at ambient temperatures, nanowire devices do not provide comprehensive information about their electronic transport. It is possible to identify distinct conduction pathways thanks to the temperature dependence of the current-voltage (*I*–*V*) properties of nanowires. The idea of impurity or Coulomb scattering and electron-phonon scattering on the surface/core of nanowires was developed after a thorough understanding of the role of carrier scattering in semiconductor nanowires.

In earlier studies, we exclusively studied the static (dc) performance of a GaN nanowire WGT at room temperature, as well as the effects of surface/core traps, architecture, and other factors [22,23,24]. Here, we have explored the effect of temperature on the gate bias-dependent carrier transport mechanism in the GaN nanowire WGT to determine how the GaN nanowire’s conductance varies. When the gate voltage was low (below the threshold voltage), the conductance decreased and subsequently increased with temperature, but when the gate voltage was high, the conductance decreased monotonically as the temperature increased. Carrier scattering mechanisms, such as electron–phonon and impurity scattering, can be used to explain these discrepancies.

## 2. Materials and Methods

We employed a GaNOI substrate from SOITEC Corporate that was manufactured using the Smart Cut^TM^ process and double-wafer transfer technology for the GaN nanowire WGT architecture. A GaN layer (150 nm thick) and buried oxide (SiO_2_, 800 nm thick) are both present on a sapphire (0.65 mm thick) substrate to form a 4-inch diameter GaNOI substrate. To begin, the crystal orientation 〈11-20〉 was etched on the GaNOI substrate using an advanced electron beam (e-beam) lithography tool and PMMA resist. The GaN film was selectively etched using an inductively coupled plasma dry etching technique, and then the device-patterned wafer was etched with a TMAH solution for 10 min at 90 °C. Instead of the vertical c-plane (0001) crystal direction, this etchant solution only etches in the lateral crystal direction. This etchant decreased the GaN film width in the crystal direction 〈11-00〉, resulting in 83 nm heights with corresponding triangular sidewall orientations 〈11-01〉. To remove the buried oxide beneath the GaN nanowires effectively, the substrate was rinsed in a buffer-oxide etchant (BOE) solution.

Additionally, on the GaN pattern, selectively develop undoped GaN (50 nm thick) and AlGaN (20 nm thick) films using the metal-organic chemical vapor deposition (MOCVD) technique. Here, the patterned GaN film served as the *r*-plane for a self-limiting re-grow process in the 〈11-01〉 orientation. It is quite impressive that the surface of the *r*-plane contains nitrogen (N) atoms that were simply interacting with hydrogen (H) atoms in the MOCVD chamber and generating N-H bonds, which enhanced stability and limited growth in the plane direction [22]. AlGaN/GaN films could so readily be formed again, though only on the source and drain regions and not on the GaN nanowire. This procedure prevented the GaN nanowire’s area from changing. Re-grown AlGaN/GaN films are crucial in this situation because they reduce series resistance by utilizing the two-dimensional electron gas (2DEG) region at the interface to the source/drain sections.

With the help of the plasma-enhanced atomic layer deposition (PE-ALD) technique, gate metal (TiN, 10 nm thick) and high-k gate oxide (Al_2_O_3_, 20 nm thick) were gradually coated for the construction of WGT devices. As a result, a four-layer metallization scheme (Ti/Al//Ni/Au) was deposited as source/drain regions using the e-beam approach, followed by rapid thermal annealing in a N_2_ environment at 850 °C for 30 s. Finally, a gate metal layer made of Ni/Au was coated to serve as an external contact for electrical properties. Using a Hall-effect measurement device (HL5500PC, Nanometrics), the concentration and mobility of the regrown AlGaN film, 9.75 × 10^12^ cm^−2^ and 1630 cm^2^/V∙s, were assessed. A field-emission tunneling electron microscope (FE-TEM, JEM-2100F) analysis of the device architecture was performed. A Keithley source (4200-SCS) linked to an MST-6VC vacuum chamber with a low-temperature control system was used to measure the device’s temperature-dependent *I–V* properties. The temperature control system has a sensitivity of ±1 K.

## 3. Results

**Figure 1:**
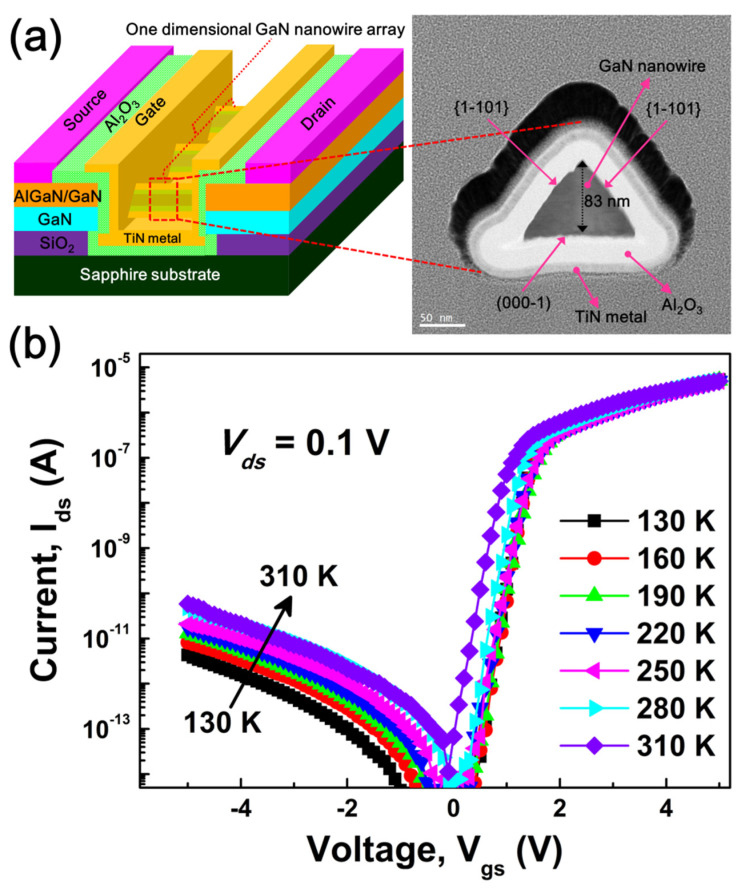
(**a**) Schematic device architecture of the fabricated GaN nanowire WGT with a high-resolution FE-TEM cross-section image of a triangular-shaped GaN nanowire. (**b**) Logarithmic plots of drain current (*I*_ds_) versus gate voltage (*V*_gs_) at *V*_ds_ = 0.1 V as a function of temperature.

The investigated GaN nanowire WGT device’s schematic architecture is shown in Figure 1a (on the left). It has a gate length of 2 µm and 64 triangular-shaped one-dimensional nanowires with identical 〈1-101〉 crystal facets on each of its two faces. The right side of Figure 1a displays a crystal-clear FE-TEM image of a triangular-shaped GaN nanowire core surrounded by gate oxide and gate metal. Figure 1b displays the drain current (*I*_ds_) vs. gate voltage (*V*_gs_) curves of the AlGaN/GaN-based GaN nanowire WGT as a function of temperature, with increments of 30 K between 130 and 310 K for the drain voltage (*V*_ds_) of 0.1 V. As can be seen, the drain leakage current demonstrates a distinct temperature dependence, rising from 1.12 × 10^−13^ A at 130 K to 2.15 × 10^−12^ A at 310 K. The increase in drain leakage current might be driven by surface-related traps and tunneling mechanisms that are facilitated by temperature [25,26].

**Figure 2:**
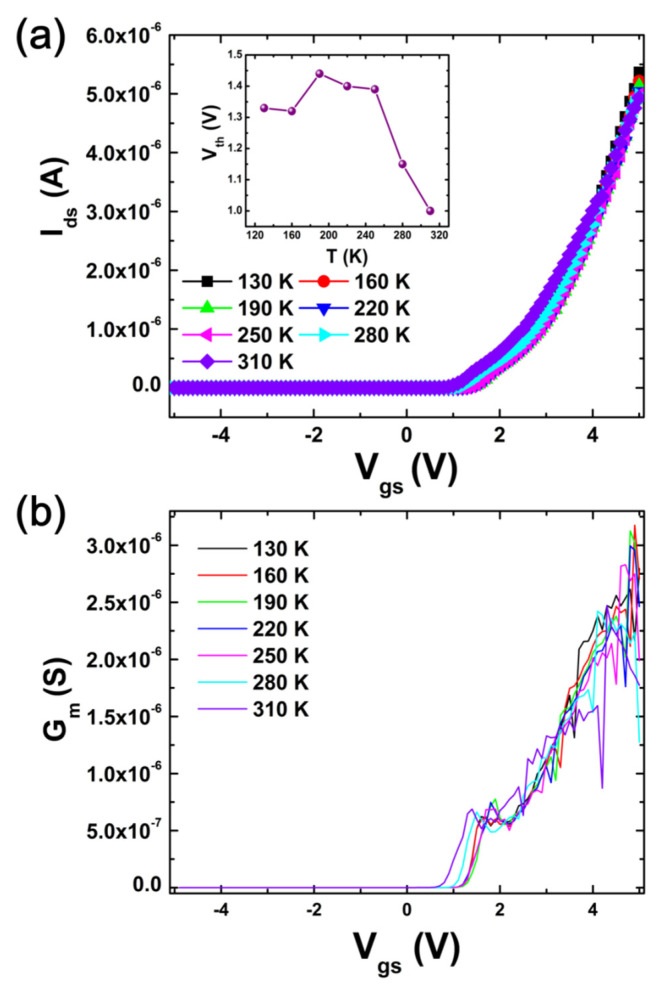
(**a**) Linear *I*_ds_ versus *V*_gs_ as a function of temperature and inset shows temperature dependence of threshold voltage; (**b**) *G*_m_ versus *V*_gs_ as function of temperature of the GaN nanowire WGT.

The linear properties of *I*_ds_ versus *V*_gs_ of GaN nanowire WGT as a function of temperature are depicted in Figure 2a. GaN WGT operates typically with a positive threshold voltage (*V*_th_) and a maximum drain current (*I*_d_,_max_) of 6 µA at all temperatures. The inset of Figure 2a depicts how *V*_th_ changes as a function of temperature. The *V*_th_ reduces with temperature in general, but a small variation reveals that the *V*_th_ declines up to the critical temperature (i.e., 160 K), reaches its maximum value at 190 K, and then rapidly decreases with temperature. Further research in the carrier transport of GaN nanowires is required because the existence of the critical temperature is not currently well known. It is observed that when the temperature rises, the total *V*_th_ gradually decreases. This may be because the doping concentration in the nanowire channel decreases due to incomplete dopant ionization at low temperatures, where it exponentially decays and becomes smaller than the electron concentration [27].

The device’s *G*_m_–*V*_gs_ properties are depicted in Figure 2b as a function of temperature. For all temperatures, the *G*_m_ increases with rising gate voltages, but there is a little variance at low gate voltages (1.4~1.9 V). This is due to the fact that surface channels open at specific gate voltages, such as 2 to 5 V, and that core channels open at low gate voltages (i.e., 1.4~1.9 V) [23]. Finally, surface channels become dominant at high gate voltages [23].

**Figure 3:**
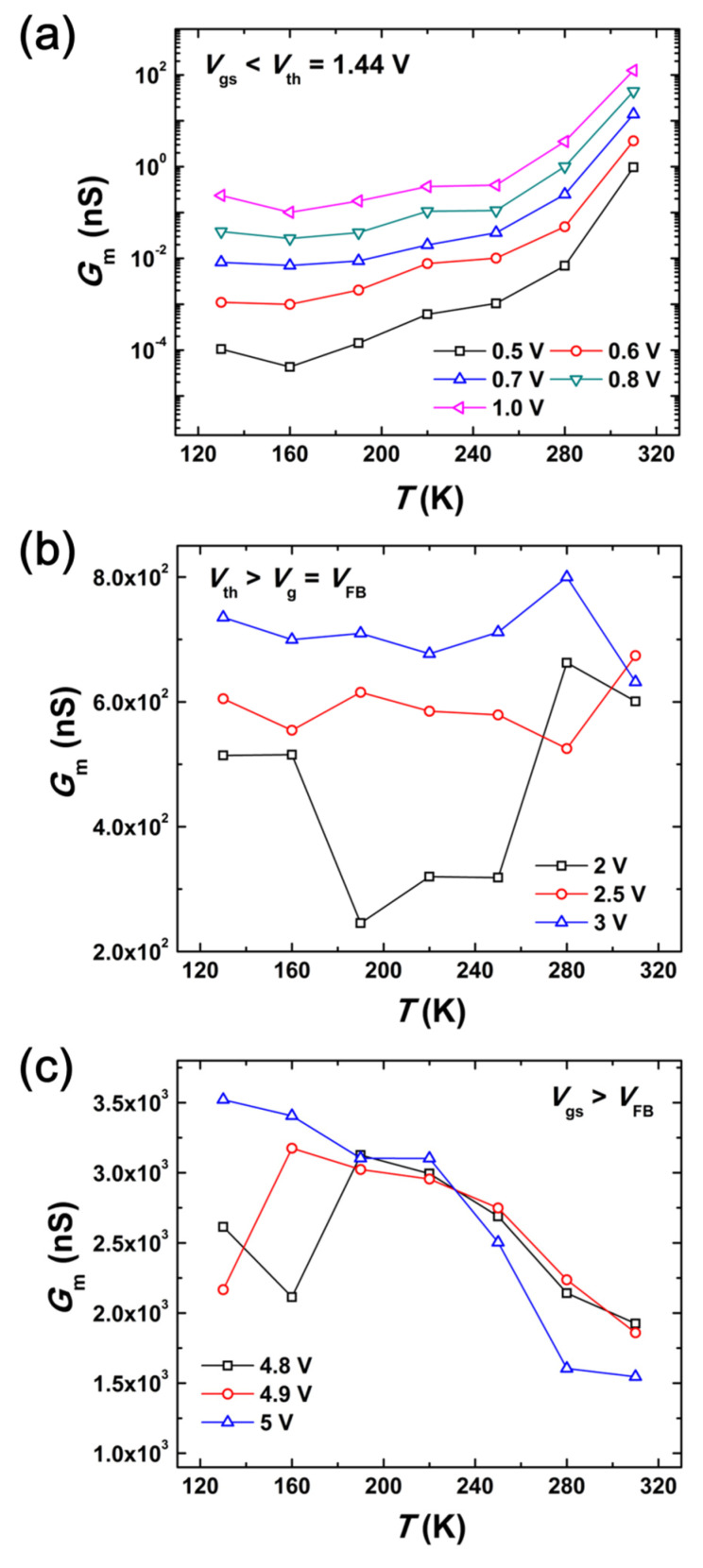
Gate voltage-dependent *G*_m_ versus *T* characteristics of the GaN nanowire WGT: (**a**) below threshold voltage; (**b**) between threshold voltage and flat band voltage; (**c**) above flat band voltage.

Using the conductance data on various gate biases could more precisely analyzes the carrier transport process across the GaN nanowires. Notably, the *G*_m_ is falling while the *V*_gs_ is falling in Figure 3. This is brought on by the nanowire channel’s carrier depletion at lower *V*_gs_. At gate bias of *V*_gs_ < *V*_th_ = 1.44 V (Figure 3a), the *G*_m_ decreases and then increases with increasing temperature; at *V*_th_ > *V*_gs_ = *V*_FB_ (i.e., *V*_FB_ = 3.7 V was evaluated from the capacitance-voltage characteristics of GaN nanowire WGT not shown here); and finally, at gate bias of *V*_gs_ > *V*_FB_ (Figure 3c), the *G*_m_ abruptly decreases with increasing temperature.

The carrier scattering mechanisms on the surface/core of the GaN nanowires can explain these characteristics. The values of *G*_m_ are high at 130 K and reach their lowest point at the critical temperature (160 K) for gate voltages of *V*_gs_ < *V*_th_ = 1.44 V. Thereafter, they grow quickly with rising temperatures. For the temperature-dependent conductance experiments, InAs nanowires showed a similar critical temperature (140 K) [19]. The typical response of the donor activation process to temperature is an increase in *G*_m_ with rising temperature. As the temperature drops, the donors become frozen, reflecting a fall in *G*_m_. *G*_m_(T) = σ_C_(T)(*A*/*L*_g_), where σ_C_(T) is the temperature-dependent carrier conductivity, *L*_g_ is the gate length, and *A* is the nanowire cross section that can be used to indicate the temperature dependence of nanowire conductance. The temperature dependency of conductivity is represented as σ_C_(T) = [*q*^2^*n*(*T*)*τ*(*T*)/m*], where *q* is the charge, *n*(*T*) is the temperature-dependent carrier density of GaN nanowire channels, m* is effective mass, and *τ*(*T*) is the relaxation time as a function of temperature. Here, the main kind of scattering mechanism is determined by the temperature-dependent relaxation time. In actuality, electron-phonon scattering is less likely when the phonons are frozen at low temperatures.

On the other hand, the data in Figure 3a demonstrates that conductance rises with applied *V*_gs_ (carrier density). The rise in conductance is normal behavior for a charged impurity scattering dominant situation. At this point, as conductivity rises, free carrier screening is intensified due to Coulombic (scattering) perturbation, which causes scattering time (also known as relaxation time, *τ(T)*) to rise with carrier density. Defects, charge dislocations, and background contaminants all contribute to the potential for Coulombic scattering. Another factor could be the dual-wafer transfer method utilized to create the epitaxial GaNOI structure, which has the potential to reduce the crystal quality of the GaN layer [22].

The conductance fluctuates after the threshold voltage and up the flat band voltage (Figure 3b, *V*_th_ > *V*_gs_ = *V*_FB_). This is a result of the complex temperature dependence of the product *n*(*T*)*τ*(*T*) caused by the interaction of charged impurities and phonon scattering, which causes a complex signature to occur in the conductivity. This suggests that surface donors in the GaN nanowire channel compensate for the core donors. Carriers remain fluctuating (i.e., *V*_gs_ = 4.8 and 4.9 V) at low temperatures, but rapidly drop with increasing temperature as *V*_gs_ > *V*_FB_ (Figure 3c). It is important to note that at *V*_gs_ = 5 V, conductance rapidly declines without any issues.

Here, we need to think about two things: (i) low conductivity is shown in the relaxation time’s sharp reduction with rising *V*_gs_ [23] and (ii) mobility reduces with rising temperature (i.e., inversely with resistance) [19]. These may be typical behaviors of the electron–phonon scattering contribution. Ultimately, we have come to the conclusion that optical electron–phonon scattering is possible at the nanowires’ surfaces and that impurity scattering is possible in the core of GaN nanowires.

## 4. Conclusions

In summary, applied gate potentials were used to investigate the electrical characteristics and carrier transport mechanism in the surface/core of GaN nanowire WGTs. When determining the carrier scattering mechanisms on the surface/core of a GaN nanowire, fundamental properties such as *G*_m_ are crucial. The *G*_m_ increase with temperature at *V*_gs_ < *V*_th_ = 1.44 V was caused by impurity scattering that possibly occurred inside the GaN nanowire’s core. At a gate bias of *V*_th_ > *V*_gs_ = *V*_FB_, the values of *G*_m_ fluctuate because the core and surface currents in GaN nanowires balance one another. Then, *G*_m_ drops with temperature, possibly because of phonon scattering, which is important at the gate bias of V_gs_ > *V*_FB_.

## Data Availability

The data is available on reasonable request from the corresponding author.
